# Push‐out bond strength of the calcium silicate‐based endodontic cements in the presence of blood: A systematic review and meta‐analysis of in vitro studies

**DOI:** 10.1002/cre2.546

**Published:** 2022-02-27

**Authors:** Mahdieh Alipour, Leili Faraji Gavgani, Negin Ghasemi

**Affiliations:** ^1^ Dental and Periodontal Research Center, Faculty of Dentistry Tabriz University of Medical Sciences Tabriz Iran; ^2^ Department of Statistics and Epidemiology, Faculty of Health Tabriz University of Medical Sciences Tabriz Iran; ^3^ Research Center for Evidence Based Medicine Tabriz University of Medical Sciences Tabriz Iran; ^4^ Department of Endodontics, Faculty of Dentistry Tabriz University of Medical Sciences Tabriz Iran

**Keywords:** blood contamination, calcium silicate‐based cement, meta‐analysis, mineral trioxide aggregate (MTA), push‐out bond strength, systematic review

## Abstract

**Objectives:**

The push‐out bond strength (POBS) of calcium silicate‐based cements (CSCs) to the dentinal wall is considered one of the essential physical properties for clinical success. The presence of blood in the treatment area affects the POBS of these types of cement. This study aimed to evaluate the impact of blood contamination on the bond strength of CSCs and dentinal walls.

**Material and Methods:**

This systematic review was performed by searching electronic databases (MEDLINE‐PubMed, Scopus, and EMBASE) to include relevant in vitro studies published between 1992 and April 2020. Two reviewers independently evaluated the selected studies and extracted data on the type of studied CSCs, evaluated area of the teeth, sample size, the dimension of a prepared area, slice thickness, storage duration, the setting of the universal testing machine (UTM), effects of blood contamination on POBS of CSCs and their failure modes. The bond strength of evaluated CSCs in studies was used for network meta‐analysis.

**Results:**

Initial searches identified 292 articles, while only 13 articles met the inclusion criteria. Full texts of these articles were evaluated, and data extraction was performed. The effect of blood contamination on bond strength to the dentinal wall was assessed in various CSCs such as PMTA, Biodentine, and AMTA. The network meta‐analysis results showed that the bond strength of Biodentine was significantly higher than other types of cement in blood presence (*p* < .05).

**Conclusions:**

Based on the current systematic review, despite controversies among the result of the different articles and the lack of data for some CSCs like bioaggregate, it could be concluded that the bond strength of Biodentine to the dentinal wall is better than other evaluated CSCs in the presence of blood.

## 1 INTRODUCTION

Root perforations due to iatrogenic or non‐iatrogenic causes are among the most common reasons for endodontic failures, with a 9.6% prevalence. These perforations cause the artificial connection between root canal space and periodontium or oral cavity (Bhagya et al., 2018; Mente et al., 2010; VanderWeele et al., 2006). Considering the connection area, blood and/or saliva could incorporate with root perforation repair materials. Root perforation repair materials should be biocompatible, dimensionally stable, radiopaque, insoluble in the presence of tissue fluids, provide an appropriate seal, and adapt with surrounding dentinal walls (Mente et al., 2010; Saeed Rahimi et al., 2013; Shalan, 2012). In the past, indium foil, gutta‐percha, amalgam, zinc oxide, glass ionomers, and calcium hydroxide were used for the perforation treatment (Bhagya et al., 2018; Mente et al., 2010). However, nowadays, calcium silicate‐based cements (CSCs) have been used in these cases due to more reported clinical successes (Chan et al., 2020). Mineral Trioxide aggregate is the first generation of CSCs developed more than 25 years ago. The wide range of calcium silicate‐based biomaterials used in the endodontic field. These biomaterials could induce mineralization at the dentine interface in the presence of moisture such as blood and saliva.

Over time, various components are added to these biomaterials to improve their physical and clinical properties, such as setting time (Almeida et al., 2014; Chan et al., 2020; Salem Milani et al., 2013). These materials are often compared with MTA, the most common biomaterial in endodontic, because of its remarkable bond strength with the dentin (Parirokh & Torabinejad, 2010).

During root perforation treatment, CSCs directly contact or mix with blood. This blood contamination has detrimental effects on the physical properties of CSCs. Proper bond strength to dentin is one of the most prominent physical properties, which was considered in evaluating the successful clinical use of endodontic biomaterials (Rahimi et al., 2013). An ideal root perforation repairing material should not be affected by blood contamination. In addition, this material should remain in place under dislodging forces applied during restorative procedures and functional.

Therefore, many studies focus on the bond strength of these materials in the presence of blood for clinical application (Lotfi et al., 2014; Ratih & Putri, 2017; VanderWeele et al., 2006).

In other words, these biomaterials should keep their proper bonding to the dentinal walls and not displace for providing a proper seal. Biomaterials bond strength with dentin is considered essential criteria for sufficient sealing ability. Insufficient seal causes the leakage of the bacteria and their products, which could lead to treatment failure (Akcay et al., 2016; Lotfi et al., 2013; Roberts et al., 2008).

The push‐out test is a standard method for assessing the bond strength. This test measures the interfacial shear strength between two surfaces and shows their adhesive properties and resistance to dislodgement (Collares et al., 2016).

Despite several studies conducted on the effects of blood contamination on the push‐out bond strength of CSCs, this systematic review was undertaken to provide comprehensive information about the impact of blood contamination on bond strength between CSCs and dentinal walls.

## 2 METHODS

### 2.1 Search strategy

PubMed, Scopus, and Web of Science databases were searched for related studies between 1992 and April 2020. The searches were done based on the modified search strategies due to different databases. The used searching terms were including Push‐out bond OR Push‐out test OR Push‐out strength OR Dislocation resistance AND Calcium silicate‐based cement OR CSCs OR Mineral trioxide aggregate OR MTA OR Biodentine OR Bioaggregate AND Blood contamination.

### 2.2 Study selection and eligibility criteria

The duplicated data from the three databases were removed. The abstract of all published articles was reviewed and presented as a flowchart. Two reviewers independently evaluated the titles and abstracts of all articles against the below inclusion and exclusion criteria.

Inclusion criteria:

− Abstract and full text available in English.
− In vitro studies, which evaluated push out bond strength of at least one CSC to the dentinal wall in the presence of blood.

Exclusion criteria:

− Incomplete data, which were not accessible by contacting the authors.
− Letters to the editor, presentations in conferences, case reports, and unpublished papers.
− Studies which were evaluated Calcium silicate, based sealers.
− Studies which were evaluated other mechanical features such as shear bond strength, microhardness, and …
− Studies that did not evaluate the bond strength of CSCs to dentinal wall.

### 2.3 Risk of bias assessment

The risk of bias assessment was performed based on previous modified tools to adapt to the in vitro nature of this systematic review (AlShwaimi et al., 2016; Neelakantan et al., 2018; Shalabi et al., 2019; Samiei et al. 2019). The quality assessment of studies was based on the following parameters: sample size calculation, standardization of specimens, randomization, blinding to experimental protocols, standardization of preparation protocol, and data reporting. The items were classified with a low risk of bias when there was no ambiguity. The Moderate risk of bias was used when one of the items was eliminated or indicated ambiguously. The lack of two or more than two items was demonstrated high risks of bias. Also, the statements in the manuscripts were noted in the results.

### 2.4 Data extraction

EndNoteX9 (Bld 13682) was used to manage references and results. The full text of selected articles was purchased, and data were evaluated by two reviewers and extracted regarding the following data: Name of the author/year of publication, number of samples, type of CSCs, anatomical location of samples, the diameter of the canal or perforated area, sample thickness, diameter and speed of the force rod, incubation time, number of samples per group, removal or non‐removal of the smear layer (Table 1), failure mode and the result of the articles due to significant effect of blood contamination on bond strength (Table 2). Moreover, the amounts of bond strength in MPa were extracted from articles for meta‐analysis. Requests for data not demonstrated in published articles were sent to the corresponding author three times. If no answer was received, the study was excluded from the analysis.

**Table 1:** Risk of bias assessment

Author/year	Sample size calculation	Standardization of Specimens	Randomization	Blinding	Standardization of preparation protocol	Reporting of data
Vander Weele et al./2006 (VanderWeele et al., 2006)	High Not Mentioned	High “Freshly extracted multi‐rooted maxillary and mandibular molars …”	High “The teeth were then divided into 12 experimental groups…”	High Not mentioned	Low “All steps were performed by a single operator.”	Low All outcomes reported
Aggarwal et al./2013 (Aggarwal et al., 2013)	High Not mentioned	Moderate “Freshly extracted mandibularmolars with no/minimal caries and non‐fused, divergingroots …”	High **“**The samples were divided into three major…”	High Not mentioned	High Single operator, disc preparation, and blood withdrawal were not mentioned	Low All outcomes reported
Rahimi et al./2013 (Saeed Rahimi et al., 2013)	High Not mentioned	Moderate **“**The inclusion criteria consisted of mature apices, no carious lesions, no shape or size anomalies, no fused roots, and no previous root canal treatments…”	High **“**The samples were divided into 12 groups of 15…”	High Not mentioned	High Single operator and blood withdrawal were not mentioned	Low All outcomes reported
Ustun et al./2015 (Üstün et al., 2015)	High Not mentioned	Moderate **“…**with mature apices, and no signs of any carious lesions, shape or size anomalies, fused roots, or previous root canal treatments…”	High **“**Each group was then subdivided into four…”	High Not mentioned	Moderate Single operator was not mentioned	Low All outcomes reported
Adl et al./2016 (Adl et al., 2016)	High Not mentioned	High **“**Freshly extracted human teeth, including mandibular single‐rooted premolars and maxillary anterior incisors, with intact or only small caries lesions were selected.”	Moderate “Specimens were then allocated randomly into eight groups of 15 on the basis of the…”	High Not mentioned	Moderate Single operator was not mentioned	Low All outcomes reported
Akcay et al./2016 (Akcay et al., 2016)	High Not mentioned	High **“**Forty‐eight single‐rooted maxillary central incisors with comparable dimensions were chosen …”	Moderate **“**The specimens were randomly separated into two groups	High Not mentioned	High Single operator and blood withdrawal were not mentioned	Low All outcomes reported
Ratih et al./2017 (Ratih & Putri, 2017)	High Not mentioned	High **“**A total of 30 human mandibular premolars were used …”	Moderate **“**The resected roots were randomly divided…”	High Not mentioned	Moderate Single operator was not mentioned	Low All outcomes reported
Ashofteh Yazdi et al./2017 (Yazdi et al., 2017)	High Not mentioned	High **“**Teeth with severe curvature, caries, cracks, and resorptive defects in the roots were excluded…”	Moderate **“**Obtained specimens were randomly divided …”	High Not mentioned	Moderate Single operator was not mentioned	Low All outcomes reported
Bhagya et al./2018 (Bhagya et al., 2018)	High Not mentioned	High **“**80 single‐rooted caries‐free human canines were selected for the study. Teeth with cracks and with any resorptive defects were excluded…”	High **“…**wasmixed in 10 samples, …	High Not mentioned	High Single operator and disc preparation were not mentioned	Moderate Results of bond failure analyzes were not reported
Marquezan et al./2018 (Marquezan et al., 2018)	High Not mentioned	High **“**Twenty‐five extracted single‐rooted human teeth with straight roots, and no root canal treatment were selected…”	Moderate “The preparedslices were randomized for …”	High Not mentioned	Moderate Single operator was not mentioned	Low All outcomes reported
Singla et al./2018 (Singla et al., 2018)	High Not mentioned	High **“**Teeth free of root caries, no endodontic treatment, and free of cracks were included …”	Moderate “The samples were randomly divided …’	High Not mentioned	High Single operator, disc preparation, and blood withdrawal were not mentioned	Low All outcomes reported
Adl et al./2019 (Adl et al., 2019)	High Not mentioned	Moderate **“**All selected teeth had mature apices with no signs of cracks, carious lesions, fused roots, size or shape anomalies, or previous root canal treatments…”	Moderate **“**The samples were randomly divided into…”	High Not mentioned	Low “All procedural steps were performed by a single operator” And “The blood was provided by one of the researchers.”	Low All outcomes reported
Shalabi et al./2019 (Shalabi et al., 2019)	High Not mentioned	High **“**Eighty single‐rooted sound human maxillary teeth extracted…”	Moderate **“**Teeth were then randomly divided into…”	High Not mentioned	Moderate Single operator was not mentioned	Low All outcomes reported

**Table 2:** Basic characteristics of the included studies

Author/year	Type of CSCs	Evaluated area	*N*	Dimension of prepared area (mm)	Slice thickness (mm)	Incubation duration (days)	Plunger dimension (mm)	Load velocity (mm/min)	Main results	Failure mode
Vander Weele et al./2006 (VanderWeele et al., 2006)	PMTA	Furcation perforation	10	1.6	2.5	1, 3, 7	‐	0.2	Blood contamination: Sig, decrease the bond strength Time: Sig, 7 days > 3 days > 1 day Liquids mixed with powder: Not Sig, DW = 2% LA = Normal saline	‐
Aggarwal et al./2013 (Aggarwal et al., 2013)	PMTA, MTA Plus, BD	Furcation perforation	10	‐	‐	1, 7	1	0.5	Blood contamination: PMTA and MTA plus: Sig, decrease bond strength/BD: Not Sig‐Time: Sig, 7 days > 1day	‐
Rahimi et al./2013 (Saeed Rahimi et al., 2013)	PMTA, CEM cement	Furcation perforation	15	1.3	2	1, 3, 7	1.1	0.5	Blood Contamination: Sig, decrease the bond strength‐Biomaterials: PMTA = CEM cement Time: Sig, 7 days > 3 days > 1 day	Mixed
Ustun et al./2015 (Üstün et al., 2015)	PMTA, Retro MTA, Supra MTA, BD	Furcation perforation	12	1.3	2	14	1	1	Blood contamination: Not Sig Biomaterials: PMTA = Retro MTA > BD = Supra MTA	Mainly adhesive
Adl et al./2016 (Adl et al., 2016)	AMTA, CEM cement	Mid root slice	15	1.3	1.2±0.05	3, 21	0.7	1	Blood contamination: Not Sig Biomaterials: MTA > CEM cement Time: Sig, 21 days > 3 days	Mainly mixed
Akcay et al./2016 (Akcay et al., 2016)	MTA(Cerkamed), BD	Apical root slice	12	‐	2	7	0.5	0.5	Blood contamination: Sig, decrease the bond strength Biomaterials: BD > MTA	Mainly adhesive
Ratih et al./2017 (Ratih & Putri, 2017)	PMTA	Mid root slice	5	1.3	3	3	1.5	1	Blood contamination: Sig, decrease the bond strength Liquids mixed with powder: Blood Contaminated group: Sig, 5% Cacl_2_ > DW = 2%LA/Not blood contaminated group: not Sig, Cacl_2_ = DW = 2%LA	Mainly cohesive and mixed
Ashofteh Yazdi et al./2017 (Yazdi et al., 2017)	PMTA, CEM cement, BD, ERRM (putty)	Mid root slice	24	1.3	1	1, 3	1	0.5	Blood contamination: Sig, increase bond strength Biomaterials: BD > ERRM = CEM cement=PMTA Time: BD and CEM cement: Not Sig/PMTA and ERRM: Sig, 3days > 1day	Mainly adhesive
Bhagya et al./2018 (Bhagya et al., 2018)	PMTA, GIC, BD, Cavit	Mid root slice	10	‐	1	‐	‐	1	Blood contamination: Not Sig Biomaterials: BD > PMTA = GIC = Cavit	‐
Marquezan et al./2018 (Marquezan et al., 2018)	AMTA, BD	Mid root slice	10	1.3	1	1, 7, 28	0.85	1	Blood contamination: AMTA: Sig, increase bond strength/BD: Not Sig Biomaterials: BD > AMTA Time: AMTA: Sig, 28 days > 7 days > 1 day/BD: Not Sig	AMTA: Mainly cohesive/BD: blood contaminated: mainly mixed, not blood contaminated: mainly cohesive
Singla et al./2018 (Singla et al., 2018)	AMTA, BD, HA, GI	Furcation perforation	15	‐	‐	1	‐	1	Blood contamination: Glass Ionomer Type II cement, Hydroxyapatite, and AMTA: Sig, decrease bond strength/BD: Sig, increase bond strength	‐
Adl et al./2019 (Adl et al., 2019)	PMTA, BD, EndoSeal MTA,	Furcation perforation	15	1.3	2	1, 7	1	1	Blood contamination:EndoSeal MTA: Not Sig/PMTA and BD: Sig, decrease the bond strength Biomaterials: PMtA = BD > EndoSeal MTA‐Time: Blood Contaminated group: Not Sig/Not Blood Contaminated: PMTA: Sig, increase bond strength, BD and EndoSeal MTA: Sig, decrease the bond strength	PMTA and BD: Mainly MixedEndoSeal MTA: Mainly Cohesive
Shalabi et al./2019 (Shalabi et al., 2019)	BD	Apical root slice	40	1	2	30	0.7	1	Blood contamination: Sig, increase bond strength Apatite nucleation: Sig, decrease in the presence of blood contamination	Blood contaminated: mainly adhesive/not blood contaminated: mainly cohesive and mixed

Abbreviations: AMTA, MTA angelus; BD, biodentine; CEM cement, calcium‐enriched mixture cement; CSC, calcium silicate cement; DW, distilled water; ERRM, endosequence root repair; GIC, glass ionomer cement; HA, hydroxyapatite; LA, lidocaine; PMTA, ProRoot MTA.

### 2.5 Statistical analysis

Studies that reported mean ± SD of push‐out bond strength in MPa were evaluated using network meta‐analysis. The amounts of bond strengths were considered by GeMTC. The random‐effects model was used for Network Meta‐Analysis and comparison of bond strength of different CSCs. For each type of cement, a forest plot was used for the graphical representation of the results. The level of confidence interval (CI) was 95%.

## 3 RESULTS

### 3.1 Search results

**Figure 1:**
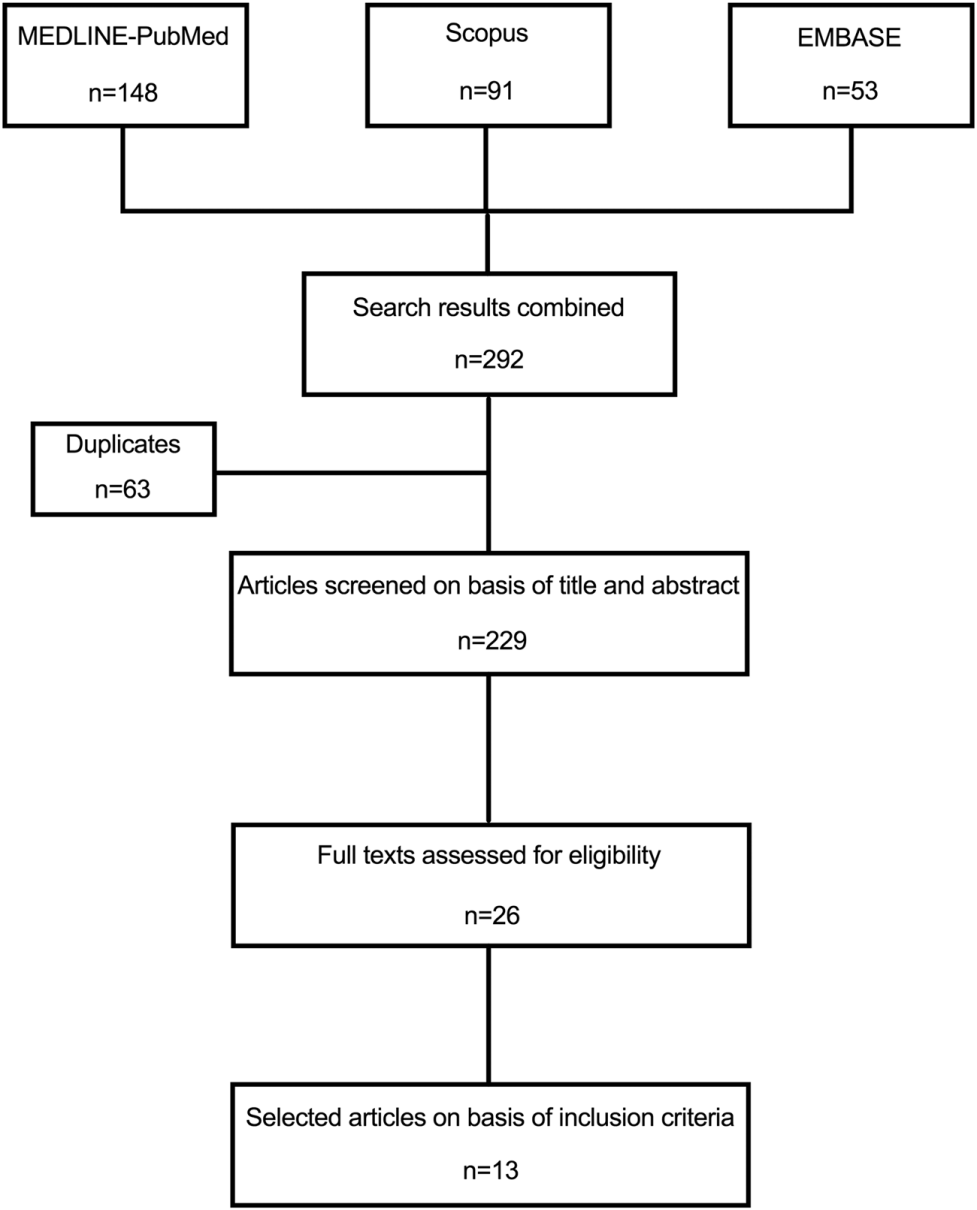
Flow chart of study selection

The initial search results from each database and progression through the search are demonstrated in Figure 1.

Seven studies were excluded because of evaluating the effects of blood contamination on cylindrical molds of CSCs without using tooth samples (Adl et al., 2015; Bolhari et al., 2020; Nekoofar et al., 2010; Oloomi et al., 2013; Shalan, 2012; Sheykhrezae et al., 2018; Singla et al., 2018; Subramanyam & Vasantharajan, 2017). Also, there were two excluded studies, which their full texts were not available in English (Park et al., 2016; Rahimi et al., 2019). Finally, thirteen studies fulfilled the inclusion criteria and were evaluated and reviewed independently by two authors.

### 3.2 Risk of bias assessment

The quality assessment of manuscripts was demonstrated in Table 1. The risk of bias was high in all of the manuscripts for sample size calculation and blinding because of the lack of details. None of the evaluated studies used the randomization assignment tool. Only two studies demonstrated disc preparation with a single operator and blood withdrawn procedures (Adl et al., 2019; VanderWeele et al., 2006). The risk of bias assessment for data reporting showed the lack of bond failure results in the Bhagya et al. study despite descriptions in the methods and materials (Bhagya et al., 2018).

### 3.3 Study characteristics

Studies have been evaluated the effect of blood exposure on the bond strength of CSCs to the dentinal wall since 2006. The extracted data on the type of studied CSCs, evaluated area of the teeth, sample size, the dimension of a prepared area, slice thickness, storage duration, the setting of the universal testing machine (UTM) (Plunger dimension and load velocity), main results and failure modes were demonstrated in Table 2.

#### 3.3.1 Type of CSCs

ProRoot MTA (PMTA), Biodentine, MTA Angelus, and CEM cement were the most evaluated CSCs in studies, respectively. Moreover, the bond strength of some of the new types of CSCs (EndoSeal MTA, Supra MTA, and MTA Plus) to the dentinal wall was evaluated in three studies (Adl et al., 2019; Aggarwal et al., 2013; Üstün et al., 2015). However, there was a lack of some CSCs such as Bioaggregate in these studies.

#### 3.3.2 Evaluated area

Furcation perforation in molars was the most evaluated area due to the high prevalence of perforation in this region during root canal treatments or post‐space preparation (Arens & Torabinejad, 1996). Apical root slices were filled with CSCs as root‐end filling material in two studies to stimulate apicoectomy clinical situations in the blood presentation (Akcay et al., 2016; Shalabi et al., 2019).

#### 3.3.3 Sample size

None of the evaluated studies demonstrate sample size calculations clearly. The evaluated dentin slices in each group ranged between five (Ratih & Putri, 2017) and forty (Shalabi et al., 2019).

#### 3.3.4 Sample preparation for the push‐out test

Seven studies used the standard diameter of 1.3 mm for the perforated area (Adl et al., 2016, 2019; Marquezan et al., 2018; Rahimi et al., 2013; Ratih & Putri, 2017; Üstün et al., 2015; Yazdi et al., 2017).

The slice thickness ranged between 1 and 3 mm in reviewed studies. Only two studies did not mention both the dimension of the prepared area and slice thickness (Aggarwal et al., 2013; Singla et al., 2018).

The incubation duration of samples differed from 1 day to 30 days. The major reviewed articles evaluated the bond strength at different periods. Most of these studies reported significant increases in bond strength of CSCs by time passing (Adl et al., 2016; Aggarwal et al., 2013; Marquezan et al., 2018; Rahimi et al., 2013; VanderWeele et al., 2006).

#### 3.3.5 The setting of UTM

Only three studies did not mention the plunger dimension (Bhagya et al., 2018; Singla et al., 2018; VanderWeele et al., 2006). The plunger dimension ranged between 0.5 and 1.5 mm, and the range of load velocity was between 0.2 and 1 mm/min.

#### 3.3.6 Effects of blood contamination on push‐out bond strength of CSCs

Eight studies (Adl et al., 2019; Aggarwal et al., 2013; Bhagya et al., 2018; Rahimi et al., 2013; Ratih & Putri, 2017; Üstün et al., 2015; VanderWeele et al., 2006; Yazdi et al., 2017) evaluated the bond strength of PMTA to the dentinal wall in the presence of blood, and five of them reported significant decreases in bond strength of PMTA after blood exposure (Adl et al., 2019; Aggarwal et al., 2013; Rahimi et al., 2013; Ratih & Putri, 2017; VanderWeele et al., 2006). Two studies reported no significant effects of blood contamination on bond strength of PMTA (Bhagya et al., 2018; Üstün et al., 2015); while only one reviewed study reported the positive effect of blood contamination on bond strength of PMTA (Yazdi et al., 2017).

Nine studies evaluated the bond strength of Biodentine to the dentinal wall after blood exposure. In three studies, bond strength was notably increased after blood contamination (Shalabi et al., 2019; Singla et al., 2018; Yazdi et al., 2017); while four studies did not report any significant differences before and after blood exposure (Aggarwal et al., 2013; Bhagya et al., 2018; Marquezan et al., 2018; Üstün et al., 2015). In spite of these manuscripts, two studies reported the adverse effects of blood contamination on the bond strength of Biodentine (Adl et al., 2019; Akcay et al., 2016).

Among three studies that evaluated bond strength of AMTA, one study indicated notable decreases in bond strength (Singla et al., 2018); while Adl et al. (Adl et al., 2016) and Marquezan et al. (Marquezan et al., 2018) studies reported not significant and increased bond strength of AMTA after blood contamination, respectively.

Comparison of the bond strength of CEM cement after blood contamination was evaluated in three studies, and the results reported incompatible data as well as AMTA (Adl et al., 2016; Saeed Rahimi et al., 2013; Yazdi et al., 2017).

The bond strength of three new CSCs (Retro MTA, Supra MTA, and EndoSeal MTA) to the dentinal wall was evaluated in studies and compared with traditional CSCs. All of these types of cement were not reported any differences in their bond strength before and after blood exposure (Adl et al., 2019; Üstün et al., 2015).

#### 3.3.7 Failure modes

Nine studies determined the failure modes among 13 reviewed articles. Only four studies reported failure modes before and after blood contamination (Adl et al., 2019; Marquezan et al., 2018; Shalabi et al., 2019; Üstün et al., 2015). The differences in failure mode pattern before and after blood exposure were only reported in two studies for Biodentine (Marquezan et al., 2018; Shalabi et al., 2019). One study mainly showed adhesive failure mode for Biodentine after blood contamination (Shalabi et al., 2019); while the other reported mainly mixed failure mode (Marquezan et al., 2018). However, the primary failure mode for Biodentine in the absence of blood contamination was cohesive in both studies.

### 3.4 Meta‐analysis

**Figure 2:**
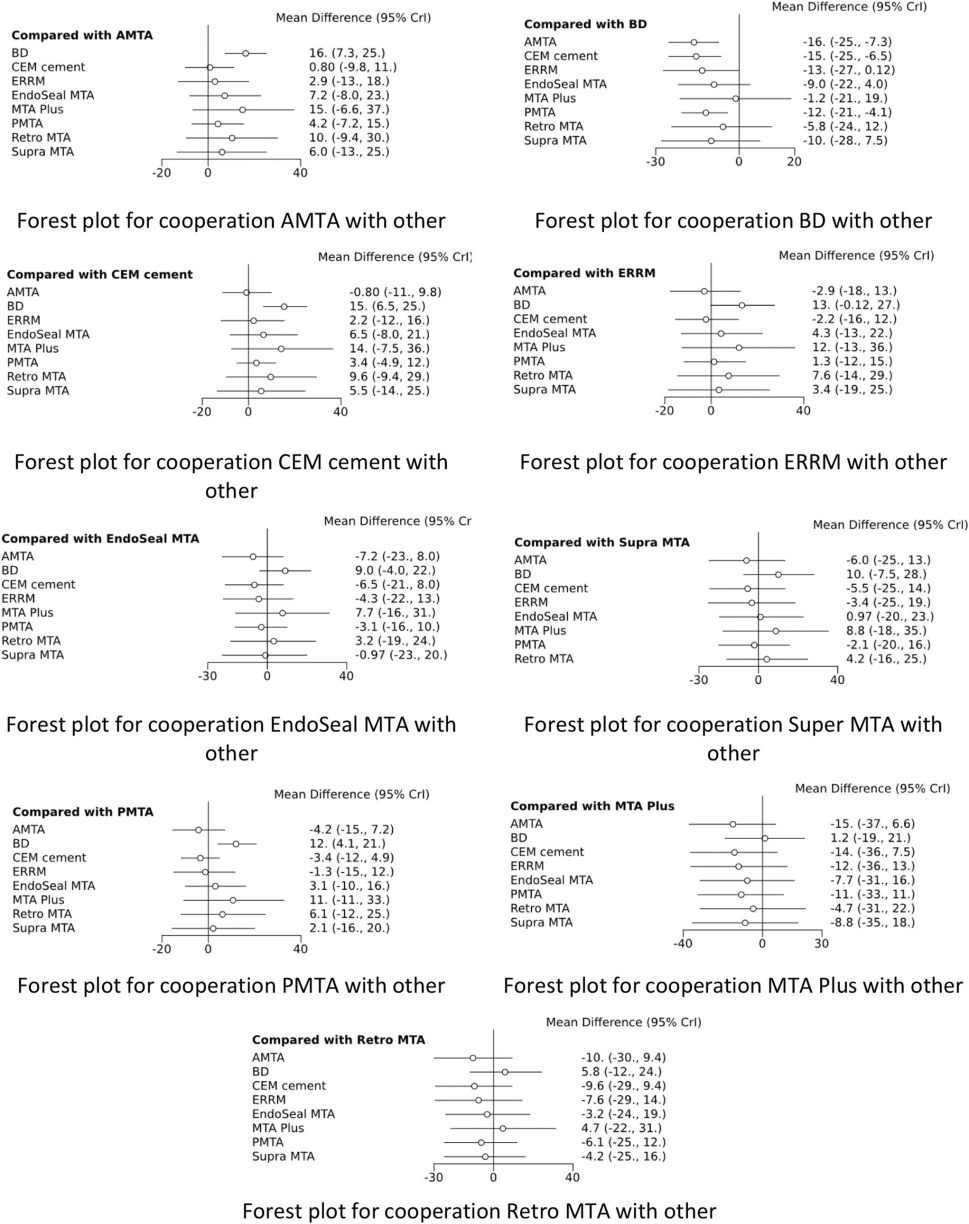
Forest plots depicting the mean difference (MD) and its 95% confidence interval for comparison types of CSCs. CSC, calcium silicate‐based cement

The bond strength of evaluated CSCs in blood presence was extracted, and network meta‐analysis was performed on the data. The network meta‐analysis results and comparison between endodontic cements were represented by the forest plot diagram in Figure 2. Based on these results, the bond strength of Biodentine in blood presence was better than other cements. The mean difference between the bond strength of Biodentine and AMTA was 26.16 (95% CI: −25% to −3.7%). Moreover, this difference was significant between BD and PMTA with −12 (95% CI: −1.4% to −21%).

## 4 DISCUSSION

This systematic review aimed to demonstrate the effects of blood contamination on the bond strength of CSCs to dentinal walls. For the push‐out bond strength test, the tensile force was applied to the longitudinal axis of the root until material dislocation. This test has an in vitro nature, which could be close to clinical situations by adding modifications. However, the results of this test could extend to clinical treatments; for example, the more bond strength indicates close contact between the material and the dentinal wall, which determined sealing ability and resistance against occlusal forces and mechanical forces of condensation of restorative materials (Adl et al., 2019; Lotfi et al., 2013; Samiei et al., 2017). CSCs increasingly were used in endodontics for vital pulp treatment, apicoectomy, and perforation treatment. Contamination with blood occurs during most of these procedures, and CSCs are applied in the presence of blood. Therefore, the properties of these materials should not be affected after exposure to the blood (Prati & Gandolfi, 2015; Singla et al., 2018). Remaining in place and providing appropriate bonds with surrounding dentin in the presence of blood is necessary for successful clinical outcomes. Different studies have been evaluating the bond strength of CSCs since introducing MTA as the first member of these types of cement (Han et al., 2015; Sluyk et al., 1998). However, few studies simulated the clinical situation in laboratory studies and evaluated the effects of blood contamination on the dislocation resistance between dentin and CSCs. After reviewing these manuscripts, the controversy in the results of these articles was identified. Moreover, significant amounts of variation in the test setup and conduction were observed. The unheterogeneity in the evaluated area and sample preparation could cause these controversies.

PMTA was the most common material in reviewed studies, which have been evaluated since 2006 in VanderWeele et al.'s study (VanderWeele et al., 2006). In most of the studies, the bond strength of this cement to the dentinal wall decreased in the presence of blood. However, the results of the three studies were in contrast to those manuscripts (Bhagya et al., 2018; Üstün et al., 2015; Yazdi et al., 2017). The differences in evaluated area, sample size, sample preparation protocols, and even setting of UTM were shown in these studies. Biodentine is another CSCs, which showed greater bond strength compared with MTA in most studies (Akcay et al., 2016; Bhagya et al., 2018; EL‐Ma'aita et al., 2013; Elnaghy, 2014; Guneser et al., 2013; Marquezan et al., 2018; Yazdi et al., 2017). Moreover, it seems that the bond strength of Biodentine is not affected by acidic PH and blood contamination (Aggarwal et al., 2013; Marquezan et al., 2018; Singla et al., 2018; Üstün et al., 2015). However, two studies showed a reduction in bond strength of this cement after exposure to blood. The results of this evaluation for three studies, which used CEM cement, are contradictory. This contradiction could happen due to different evaluating areas and incubation periods in these studies. The bond strength of three new CSCs was evaluated and compared with traditional CSCs. The results of these studies reported no differences in bond strength of these types of cement before and after exposure to the blood (Adl et al., 2019; Akcay et al., 2016).

The network meta‐analysis results confirmed the better bond strength of Biodentine compared to other ones. Based on this systematic review and network meta‐analysis, the authors suggested that Biodentine has reported a better bond than CSCs; therefore, it could be more appropriate in apicoectomy and perforation treatments. Furthermore, the new CSCs should be evaluated in future studies.

## 5 CONCLUSION

Based on the results of this systematic review, despite controversies among the result of the different manuscripts and the lack of data for some CSCs like Bioaggregate, the bond strength of Biodentine to the dentinal wall is higher than other CSCs. Therefore, Biodentine may be advantageous, especially for challenging clinical endodontic treatments such as apicoectomy and perforations in the presence of excessive blood.

## CONFLICTS OF INTEREST

The authors declare no conflicts of interests.

## AUTHOR CONTRIBUTIONS

*Writing—original draft preparation, conceptualization, methodology, data extraction*: Mahdieh Alipour. *Meta‐analysis and methodology*: Leili Faraji Gavgani. *Conceptualization, supervision, review, and editing*: Negin Ghasemi. All authors have read and agreed to the published version of the manuscript.

## Data Availability

Data sharing is not applicable to this article as no new data were created, and all analyses were reported in this study.
